# Supplementary material for: Excitations in a superconducting Coulombic energy gap
Source: Nat Commun. 2022 Apr 26;13:2243. doi: 10.1038/s41467-022-29634-5 (PMC9042936; doi:10.1038/s41467-022-29634-5)
Supplement: Supplementary file 1 — Supplementary Information [file 41467_2022_29634_MOESM1_ESM.pdf]

Excitations in a superconducting Coulombic energy gap: Supplementary Information

Juan Carlos Estrada Saldaña[1],* Alexandros Vekris[1,2], Luka Pavešić[3,4], Peter Krogstrup[1], Rok Žitko[3,4], Kasper Grove-Rasmussen[1], and Jesper Nygård[1†]

[1] *Center for Quantum Devices, Niels Bohr Institute, University of Copenhagen, 2100 Copenhagen, Denmark*
[2] *Sino-Danish College (SDC), University of Chinese Academy of Sciences*
[3] *Jožef Stefan Institute, Jamova 39, SI-1000 Ljubljana, Slovenia and*
[4] *Faculty of Mathematics and Physics, University of Ljubljana, Jadranska 19, SI-1000 Ljubljana, Slovenia*

* Corresponding author: juan.saldana@nbi.ku.dk
† Corresponding author: nygard@nbi.ku.dk

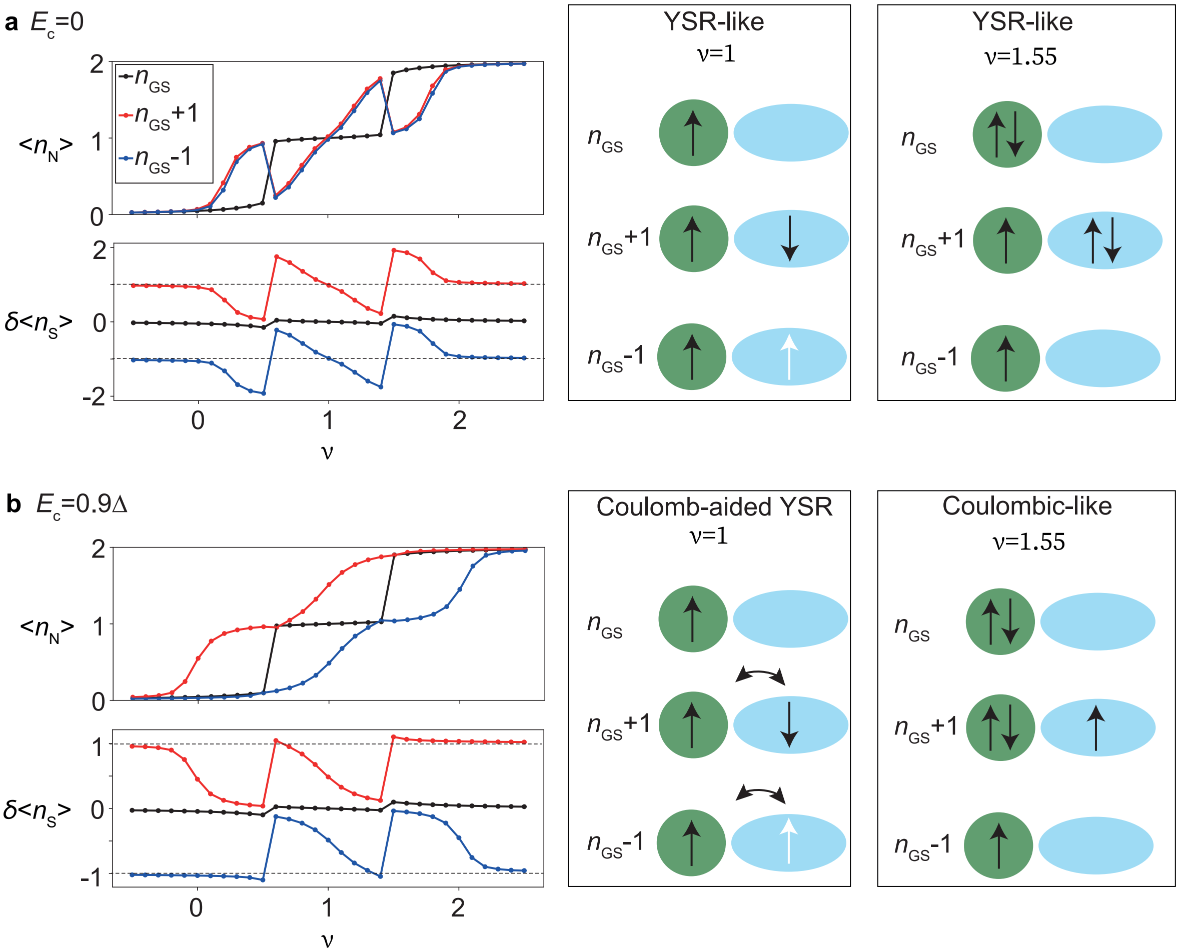

Supplementary Fig. 1. **Charge redistribution in the quantum dot and in the superconducting island.** Calculated occupation expectation values versus gate-induced charge in the QD, ν, for **a** $E_{\mathrm{c}} = 0$ and $V = 0$ (other parameters are the same as those in Table 1) and **b** device parameters in Table 1, and corresponding QD and SI filling schematics for specific ν values. The calculation is done for $N = 800$ and $n_0 = 800$. The occupation expectation values are those of the QD, $\langle n_{\mathrm{N}} \rangle$, and of the SI, $\delta\langle n_{\mathrm{S}} \rangle$, where δ indicates that the number shown is the variation in occupation over 800 electrons. The values shown correspond to the GS and the first addition and removal excitations. The addition and removal expectation values overlap in (**a**), but do not do so in (**b**), leading in the latter case to the double-S shape of the SCE. In the QD and SI charge filling schematics, black (white) arrows represent electron (hole) spins. These schematics show that, whereas at $\nu = 1$ both (**a**) and (**b**) are Yu-Shiba-Rusinov (YSR)-like with electron-hole symmetric excitation expectation values (with double-headed arrows in (**b**) reflecting the fractional expectation values for the QD and SI occupations), away from $\nu = 1$ (e.g., right after the charge fluctuation point at $\nu = 1.55$) there is a strong asymmetry in addition and removal occupation values in (**b**) not present in (**a**). The asymmetry comes from having only one level in the QD which can be filled with up to 2 electrons. As a consequence, in the $n_{\mathrm{GS}} + 1$ state an additional electron must go into the SI. In contrast, in the $n_{\mathrm{GS}} - 1$ state an electron can be removed from the SI *or* from the QD. In general, the charge excitations are electron-hole asymmetric (Coulombic-like) for $\nu \neq 1$.

Supplementary Note 1. Parameter extraction methods

Here we detail the methods which we used to extract the QD-SI device parameters shown in Table 1 of the main text. Similar methods as those outlined here and at the end of Supplementary Note 2 were used to extract the parameters of the SI-QD-SI device.

U is obtained from Coulomb diamonds spectroscopy for a fixed V_{S} such that the SI is placed in deep Coulomb blockade and only acts as a cotunneling probe of the QD (see Supplementary Fig. 2a). The measurement is performed at finite B to partially suppress superconductivity. From the length of the vertical arrow, which goes from zero bias up to the apex of the central 1,0 Coulomb diamond and which is equal to $U/2$ for asymmetric source and drain tunnel barriers, we extract $U = 0.8 - 1.0$ meV. From the ratio of $U/2$ to the V_{N} extension of the diamond, we find the lever-arm parameter of V_{N}, $\alpha_{\mathrm{N}} = 0.26$ mV/mV.

E_{c} is also estimated from Coulomb diamonds spectroscopy (shown in Supplementary Fig. 2b). V_{N} is fixed such that the QD is placed in deep Coulomb blockade acting as a cotunneling probe of the SI. Black lines show the prolongation of the equidistant (in V_{S}) conductance lines at high bias (V_{sd} outside of $\approx \pm 0.4$ mV) coming from higher excitations towards zero bias such as to form a Coulomb diamond [1]. The zero-bias crossings of this diamond correspond to $n_{\mathrm{GS}} = n_{\mathrm{GS}} + 1$ and $n_{\mathrm{GS}} + 2 = n_{\mathrm{GS}} + 3$, and are therefore spaced by $2e$. Thus, the height of the diamond is approximately $2E_{\mathrm{c}}$ (given by the vertical arrow), from which we find $E_{\mathrm{c}} \approx 0.19$ meV. This approximation ceases to be valid when E_{c} is significantly larger than Δ. From the ratio of E_{c} to the V_{S} extension of the diamond, we find the lever-arm parameter of V_{S}, $\alpha_{\mathrm{S}} = 0.32$ mV/mV.

The measurements of Γ and V are based on the curvature and spacing of the GS transition lines in the stability diagram, inspired by similar measurements in double QDs in the normal state [2]. This extraction is performed in the portion of the stability diagram magnified around the triple (TP) and quintuple (FP) points shown in Supplementary Fig. 2c. At the TP, the charge states 1,0, 1,1 and 2,0 are degenerate, while at the FP the states 1,1, 1,2, 2,2, 2,1 and 2,0 are degenerate, as corroborated from their Zeeman splitting.

To measure Γ, we first prolong with dashed lines the approximately horizontal and vertical conductance lines in the diagram of Supplementary Fig. 2c. Γ is then given by the length of the blue bars, which is the distance between the intersection of the curved conductance lines with the line joining the TP and FP, and the intersection of the dashed lines. We convert this length which is in units of gate voltage to energy by using α_{N} and α_{S}. We find $\Gamma \approx 0.05$ meV.

Second, we assume that the system behaves approximately as a double QD in the normal state in the two-electron charge sector and we equate the length of the red line to $2\Gamma + \sqrt{2}V$. Using α_{N} and α_{S}, we find $V = 0.13$ meV.

By using our model to interpret the spectral data in Fig. 3b of the main text we obtain $\Delta = 0.2$ meV, which is slightly smaller than $\Delta = 0.270$ meV of the parent Al superconductor in devices fabricated out of the same batch of nanowires [3–5], and which may be attributed to weaker hybridisation between the InAs nanowire and the Al superconductor [6], as we reported before [4].

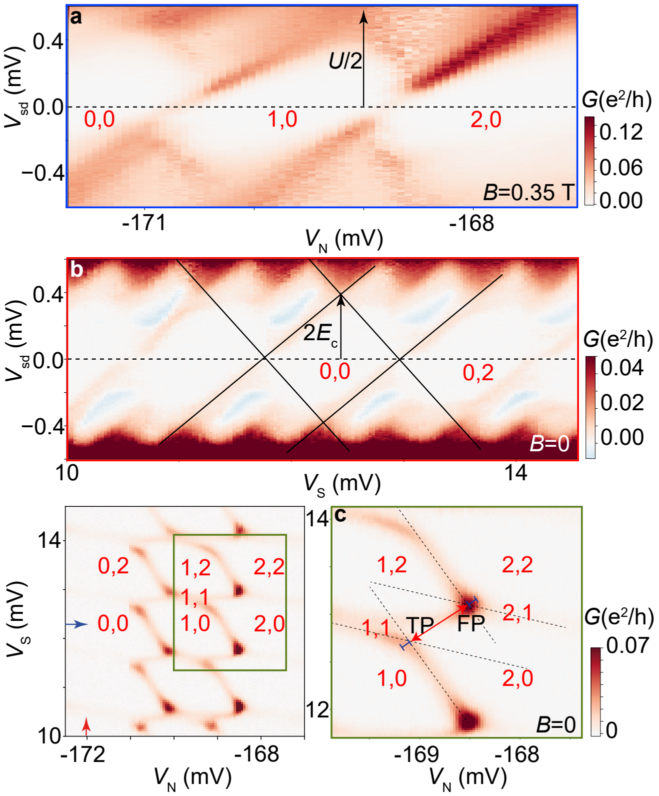

Supplementary Fig. 2. **Extraction of QD and SI parameters from experimental data.** The stability diagram from Fig. 3a is copied on the bottom left panel to illustrate with arrows and boxes the gate-sweep directions/ranges of the data in (**a**-**c**). **a** G versus V_{sd} at $B = 0.35$ T taken with V_{N} swept in the direction of the horizontal blue arrow in the stability diagram. **b** G versus V_{sd} at $B = 0$ taken with V_{S} swept in the direction of the vertical red arrow in the stability diagram. **c** Zoomed region inside the green box of the stability diagram. The meaning of the lines drawn on top of the plots is explained in the text.

The set of values is in reasonable agreement with the model parameters in Table 1 of the main text which produce the best fit to the stability diagrams and the spectra in Fig. 3 and Supplementary Fig. 4.

The effective g-factors in Table 2 of the main text are obtained from the B dependence of the stability diagram shown in Fig. 3a of the main text. To extract g_{S} we subtract the V_{S} extension of the 2,1 charge domain at $B = 0.3$ T and $B = 0$. Then, using α_{S} to convert that splitting to Zeeman energy, E_{ZS}, we find $|g_{\mathrm{S}}| = E_{\mathrm{ZS}}/\mu_{\mathrm{B}}B = 6.9$. Through a similar procedure, using instead the V_{N} extension of the 1,0 charge domain and α_{N}, we find $|g_{\mathrm{N}}| = E_{\mathrm{ZN}}/\mu_{\mathrm{B}}B = 2.9$.

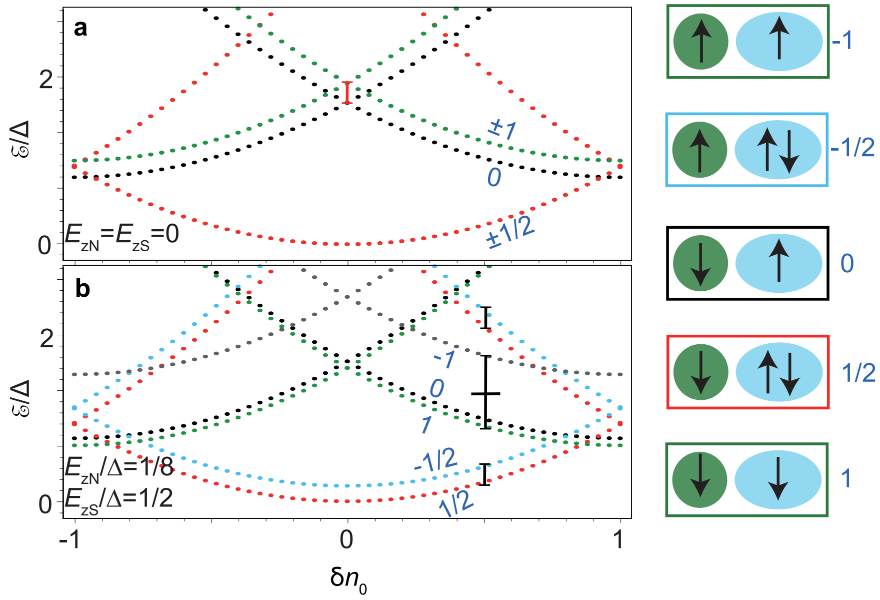

Supplementary Fig. 3. **Charge parabolas at finite** B **and exchange interaction.** (**a,b**) Calculated charge parabolas versus δn_0 at $\nu = 1$ for Zeeman energies of the QD and the SI indicated in each panel, where δn_0 corresponds to the gate-induced charge in the superconductor, n_0, shifted by $N = 200$. Each parabola is tagged by its S_z number. A red bar in (**a**) indicates the singlet-triplet exchange splitting. Black bars in (**b**) indicate doublet and triplet Zeeman splittings. The $S_z = 0$ triplet state is not included in the calculation. The sketches on the right show spin states color-coded as the states shown in (**b**) for $\delta n_0 = 1$.

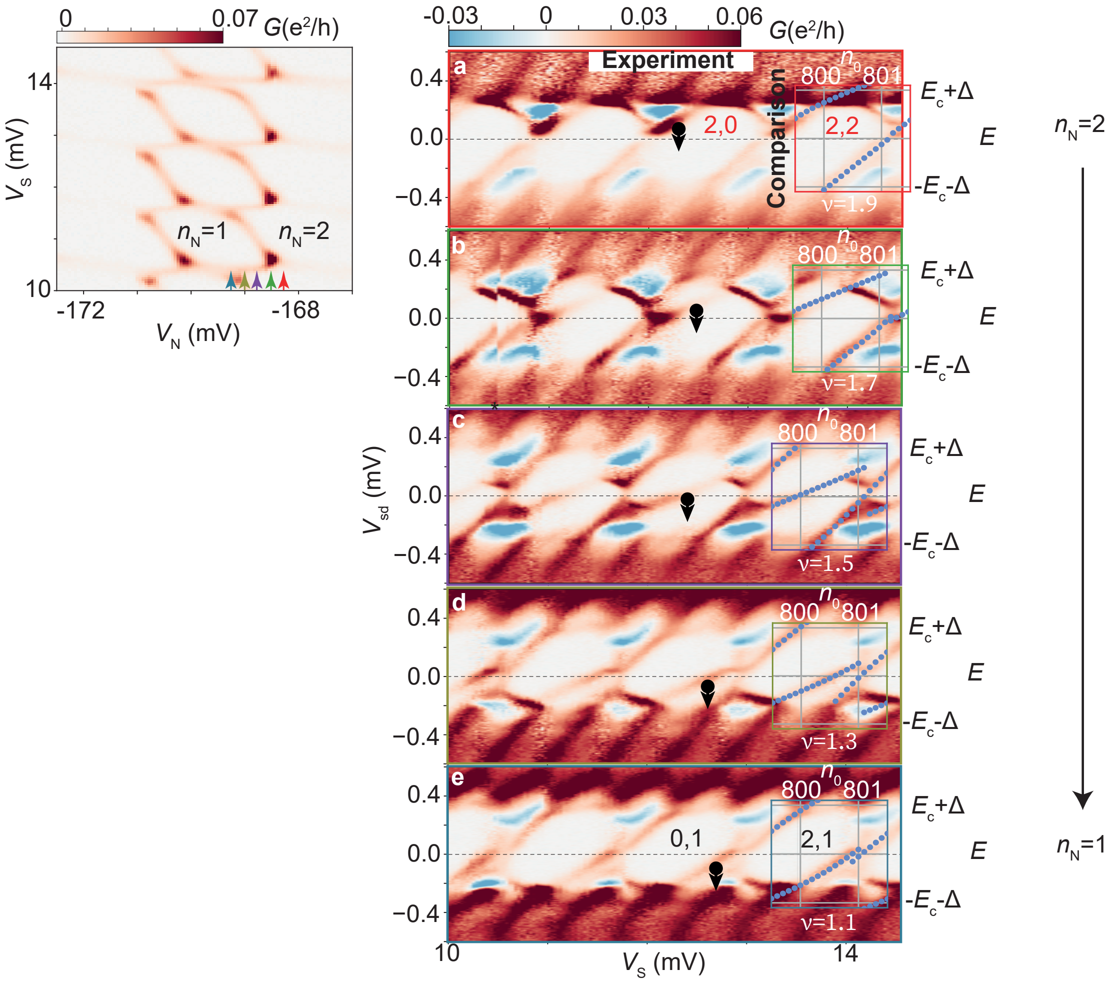

Supplementary Fig. 4. **Changes in the V_S dependence of superconducting Coulombic states across a $n_N = 2 \rightarrow 1$ transition.** (**a**-**e**) Colormaps of G versus source-drain bias, V_{sd}, and SI gate voltage, V_S, at various settings of the QD gate voltage, V_N, indicated by (**a**) red, (**b**) green, (**c**) purple, (**d**) yellow and (**e**) cyan vertical arrows in the stability diagram on the left, which is a duplicate of Fig. 3a in the main text. The tails of the black arrows are attached to a subgap state, while their heads point to the direction of the evolution of said state with varying V_N. The color scale is saturated to highlight subgap excitations. Charge numbers of the QD and the SI, n_N and n_S, are indicated in (**a**) and (**e**). An unwanted gate glitch is indicated by an asterisk in (**b**). Calculated SCE spectra using the same model parameters as in Fig. 3a in the main text are overlaid as blue dots on each panel for comparison. In the calculation, ν is fixed to the values indicated on top of each plot, and n_0 is swept. The calculation matches the position of positive-slope SCE, but does not account for negative-slope features, negative G features, and continuum features.

Supplementary Note 2. Other Γ regimes in the QD-SI device

In this Supplementary Note, we show additional data from the QD-SI device for smaller and larger Γ values than the *intermediate* Γ case in the main text. Device parameters in these other regimes are provided in Supplementary Table 1. We classify these regimes according to the following experimental considerations, which have the advantage of being model independent:

1. **Weak** Γ. The curvature of the G lines in the stability diagram is below the width of these lines. Multi-degeneracy points (e.g., TPs) are visible.

2. **Intermediate** Γ. The curvature of the G lines in the stability diagram is resolvable. Multi-degeneracy points are still visible.

3. **Strong** Γ. GS transitions due to charge addition in the QD cannot longer be distinguished. Multi-degeneracy points are no longer defined.

Supplementary Table 1. **Parameters of the QD-SI device and model for strong and weak Γ.** For each Γ strength, the top-row parameters are estimates obtained from measurements, while the bottom-row ones are obtained from best fits of the model output to the experimental data based on the measured parameters as the initial input for subsequent fine tuning. Extraction methods are shown at the end of Supplementary Note 2.

Γ strength	Γ (meV)	U (meV)	E_c (meV)	Δ (meV)	V (meV)
Strong	-	1.2	0.39	≤ 0.27	-
	0.4	0.8	0.4	0.2	0.16
Weak	≈ 0	0.9	0.21	≤ 0.27	0.24
	0.004	0.8	0.26	0.2	0.24

Weak Γ regime and indications of elastic cotunnelling

A hint of a cotunnelling transport mechanism is provided by observations of current blockade in the weak Γ regime. In sufficiently weakly coupled double QDs, a finite bias voltage leads to the formation of triangles in the current stability diagram, which arise as the two QD levels are detuned from each other within the bias window [2, 7]. Similarly, in the limit of weak Γ (Supplementary Table 1) in the QD-SI system current triangles appear at finite bias due to the gate-dependent energy difference between the GS and the discrete and continuum excitations probed in transport. Here, we report the observation of a bias-polarity dependent disappearance of the current triangles in the finite-bias stability diagram. The importance of this finding is that it hints at elastic cotunneling as a possible transport mechanism in our device and thus, at coherence over the full device.

Our model reproduces well the gate positions of conductance lines in the stability diagram, as shown in Supplementary Fig. 5b. For full agreement, the theory diagram needs to be rotated by 3.4 degrees to account for a small capacitive cross-talk between V_N and V_S. This was not needed for plots in the intermediate-Γ regime as a fine tuning of Γ could be done to compensate for this rotation. To reproduce the presence of a quadruple point (QP) in the experimental stability diagram we set $E_\mathrm{c} \approx V$, which is consistent with the experimental values of these parameters.

Three current triangles are in principle expected to emerge at finite bias out of the two TPs (solid circles) and the QP (open circle) observed at zero-bias at the convergence of the

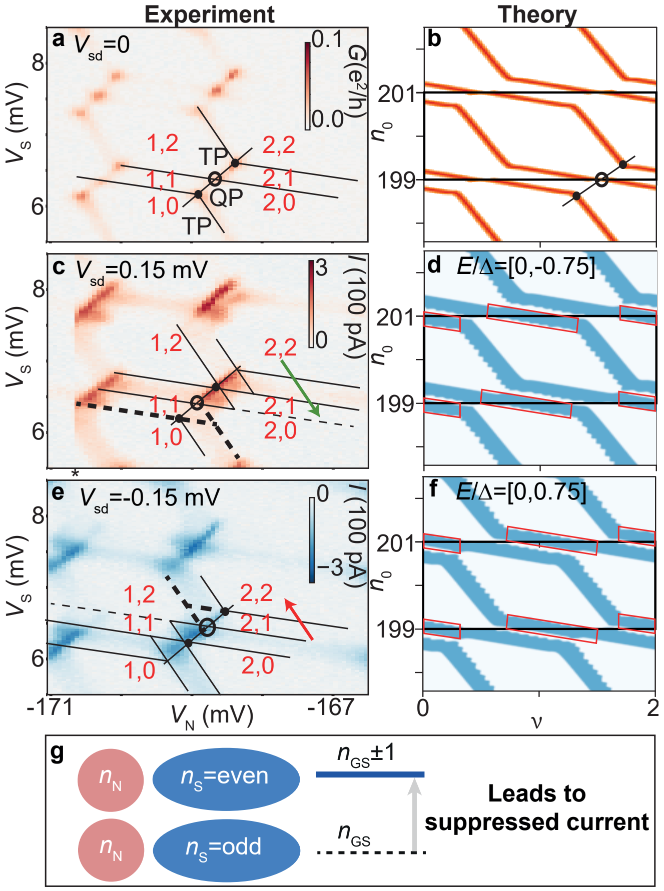

Supplementary Fig. 5. **Parity-dependent current blockade in the weak coupling regime in the QD-SI device. a** Zero-bias G stability diagram in the weak Γ regime. **b** GS crossings (represented as red lines) calculated for $N = 200$. The graph is a collage of three identical plots with n_0 ranging from 199 to 201, which are stitched copies of the plot highlighted by the rectangle with thick borders. **c**,**e** Current, I, stability diagrams measured at two opposite bias polarities, indicated in each plot. Gate settings for (**a**,**c**,**e**): $V_{\mathrm{G1}} = -350$ mV, $V_{\mathrm{G3}} = -52$ mV, and $V_{\mathrm{G5}} = -100$ mV. Note the +69 mV change in V_{G5} with respect to Fig. 3a of the main text. An asterisk in (**c**) indicates an unwanted gate glitch. **d**,**f** Calculated stability diagrams depicting excitations within the (**e**) negative and (**f**) positive ranges of E/Δ indicated in each plot, to be contrasted with experimental diagrams in (**c**) and (**e**). See text for details. Parameters of the experimental diagrams and calculations are indicated in Supplementary Table 1. **g** Sketch of the *odd→even* transition in the occupation of the SI, n_S, absent in the data in (**c**,**e**) for any parity of the occupation of the QD, n_N. The suppression of this transition leads to missing current bands and missing current triangles.

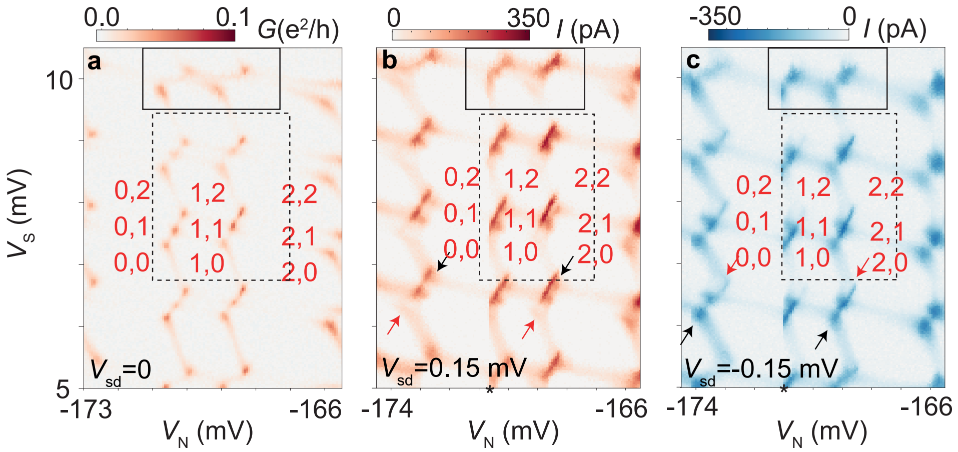

Supplementary Fig. 6. **Stability diagram in the weak coupling regime in extended gate voltage ranges.** **a** G and **b**,**c** current, I, stability diagrams at different source-drain bias voltages, V_{sd}, indicated in each plot. Asterisks indicate unwanted gate glitches. Dashed boxes indicate the gate ranges from which the magnifications in Supplementary Figs. 5a,c,e were extracted. Black arrows in (**b**) point to examples of current triangles which nearly disappear when the polarity of the bias is reversed in (**c**) (pointed then by red arrows), and vice versa.

six charge states indicated in red in Supplementary Fig. 5a [2]. However, as seen in the finite-bias current (I) diagrams in Supplementary Figs. 5c,e, one of the three triangles is always gone; i.e., only two triangles are visible per trio of two TPs and one QP. Occasionally, the base of the missing triangles remains. To guide the reader through the observed phenomena, we draw lines on top of the data in Supplementary Figs. 5a,c,e. At $V_{\mathrm{sd}} = 0$, in Supplementary Fig. 5a, we indicate the GS boundaries as continuous lines, intersecting at two TPs flanking a QP. In Supplementary Figs. 5c,e, we use continuous lines of the same slope as the zero-bias GS boundaries to identify the two visible triangles, and thick dashed lines to identify the absent triangle.

As shown in the extended diagrams in Supplementary Fig. 6, this observation is robust over several pairs of triangles, provided that Γ is weak. Intermediate Γ results in washed-out current triangles and the bias-polarity dependent current blockade is not as clearly seen; this occurs in the sections of the stability diagram within the boxes of solid contour. The presence of larger Γ here is deduced from the finite curvature of the conductance lines in the zero-bias stability diagram in Supplementary Fig. 6a.

The origin of the missing triangles resides in a *blocked transition*. In Supplementary Figs. 5d,f, we plot calculated stability diagrams in which the blue bands depict all possible excitations within the given energy range, to be compared with the experimental diagrams in Supplementary Figs. 5c,e. As positive (negative) bias corresponds to transport by emptying (filling) the QD-SI, negative (positive) ranges of energies are used. At $V_{\mathrm{sd}} = \pm 0.15$ mV, the bias window covers $\approx \mp 0.75\Delta/e$. It is apparent from the comparison that $I \approx 0$ for transitions within red boxes in the calculated diagrams. We give an example of this observation in the 2,1→2,2 transition, indicated in Supplementary Figs. 5c,e by green and red arrows, respectively. At positive bias, the 2,2→2,1 discrete transition is probed together with even→odd transitions involving excitations to the SI continuum. In this case, a band of current is observed in Supplementary Fig. 5c. However, at negative bias, the 2,1→2,2 and odd→even transitions are instead probed. The expected current band is absent in Supplementary Fig. 5e. Transitions which change the parity of the QD but keep the parity of the SI constant are instead allowed; e.g., the 1,0-2,0 transition produces a current band at both positive and negative bias. Due to this, the current is not exactly zero inside the missing current triangles.

In general, current bands are absent in Supplementary Figs. 5c,e whenever the GS corresponds to a state with *odd* occupation on the SI, and the respective discrete or continuum excitations involve a parity change of the SI from odd to even (Supplementary Fig. 5g). In the absence of a QD, a single transport path for elastic cotunneling is available in the odd-to-even parity transition of the SI, as opposed to the multiple paths available in the even-to-odd transition, which leads to a parity-dependent current suppression, as detailed in Ref. [8]. To corroborate whether this also holds for the QD-SI system, future modelling of the current is required.

Strong Γ regime

For large Γ, only parity alternation of the SI is observed, as shown in the stability diagram of Supplementary Fig. 7a, with line wiggling being the only indication of the presence of the QD. Tuning of V_{G3} in order to decrease Γ unmistakably demonstrates the presence of the QD in this regime, as shown in Supplementary Fig. 8. Supplementary Fig. 7b shows the output of our model for the GS boundaries of the stability diagram. The calculated diagram matches well the conductance lines in the experiment.

In Supplementary Fig. 7c, we show the measured SCE spectrum. The subgap excitations show *discontinuities* (indicated by arrows) when a new charge is removed from the QD-SI, as evidenced by the alignment of the discontinuities to the edge of the Coulomb diamonds corresponding to the

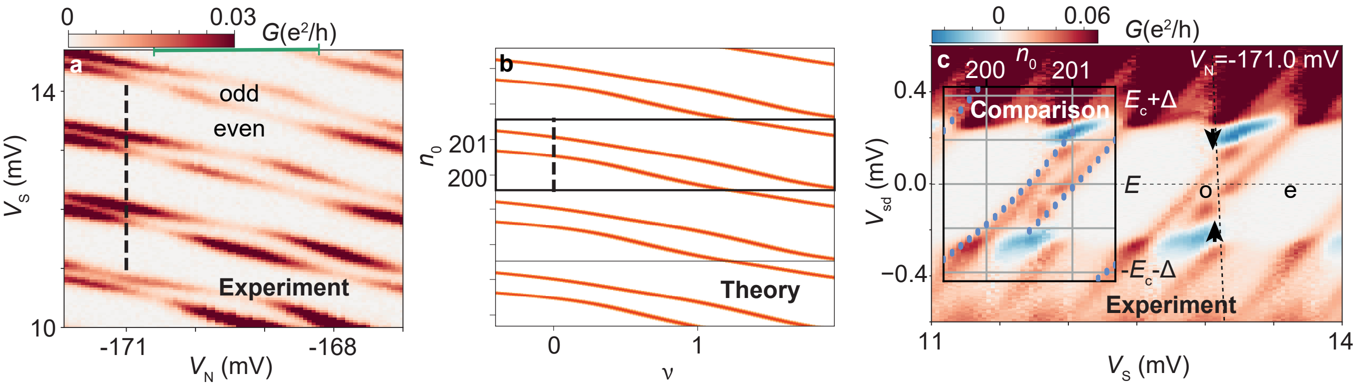

Supplementary Fig. 7. **Strong coupling regime in the QD-SI device.** **a** Zero-bias G versus $V_{\rm S}$ and $V_{\rm N}$, taken with other gates set at $V_{\rm G1} = -350$ mV, $V_{\rm G3} = -65$ mV, $V_{\rm G5} = -165$ mV. Note that $V_{\rm G3}$ has been changed by -13 mV with respect to Fig. 3a of the main text. A horizontal bar indicates $\approx U$. **b** Calculation of the GS stability diagram. The diagram is rotated by 3.4 degrees with respect to the experimental one due to $V_{\rm N}$, $V_{\rm S}$ capacitive cross-coupling. **c** Colormap of G versus $V_{\rm sd}$ taken with $V_{\rm S}$ swept along the dashed line in (**a**). Arrows point to subgap-state discontinuities. Odd (o) and even (e) sectors, which repeat themselves as the SI is filled, are indicated. The color scale is saturated to highlight faint subgap excitations. A calculation of the superconducting Coulombic spectrum (blue dots) for $\nu = 0$ (dashed line in (**b**)) is overlaid on the data after mirroring the spectrum with respect to its horizontal axis. Parameters of the experiment and calculation are given in Supplementary Table 1.

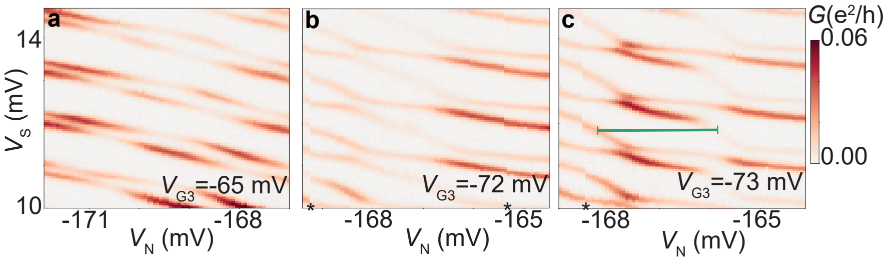

Supplementary Fig. 8. **Revelation of lurking QD in strong-coupling regime.** **a**-**c** Evolution of the stability diagram in Supplementary Fig. 7a with $V_{\rm G3}$, from (**a**) strong to (**b**, **c**) weaker Γ regimes.

SI continuum at $V_{\rm sd} \approx 0.4$ mV. To highlight this alignment, a vertical dashed line has been added at $V_{\rm S} = 13.2$ mV. At the discontinuities, the subgap excitations jump by ≈ 0.1 mV. This is a rather ubiquitous phenomenon, also present in similar measurements at different $V_{\rm N}$.

A calculation of the spectrum for $\nu = 0$ is overlaid on top of the experimental data in Supplementary Fig. 7c, after mirroring it with respect to to $E = 0$. It is found to match reasonably well the gate positions of the discrete subgap excitations; however, it fails to reproduce the observed discontinuities. As the model is not a transport model and does not account for the continuum, it also does not reproduce negative $dI/dV_{\rm sd}$ nor most of the features above $|V_{\rm sd}| \approx 0.3$ mV. Due to the relation of the discontinuities with the removal of a particle from the QD-SI, the observed discontinuities are possibly related to transport through the system (for example, to the effect of V on transport through the SI continuum), which highlights once more the need of more complex modelling.

Parameter extraction methods for weak and strong coupling

To obtain the QD-SI device parameters shown in Supplementary Table 1, we assume that the lever-arm parameters of the gates found in the intermediate Γ regime remain the same in these slightly different gate settings. In both regimes, we find $E_{\rm c}$ by measuring the distance in gate voltage $V_{\rm S}$ between the first and third topmost peaks in the stability diagrams of Supplementary Fig. 5a and Supplementary Fig. 6a. We then convert the gate voltage distance, which is equal to $2E_{\rm c}/\alpha_{\rm S}$, to energy.

In the strong Γ regime, determinations of U, Γ and V are difficult due to the nearly single-dot character of the stability diagram. To estimate U, we tune the device quasi-continuously into a less hybridized regime, as shown in in Supplementary Fig. 8. U corresponds to the green horizontal bar in Supplementary Fig. 8c, converted to energy by using $\alpha_{\rm N}$.

In the weak Γ regime, U is measured as the horizontal $V_{\rm N}$ distance between the midpoints of the vertical conductance lines delimiting the 0,1 charge sector in Supplementary Fig. 6a. The measured distance, $2U/\alpha_{\rm N}$, is then converted to energy. To determine Γ and V, we employ on the stability

diagram of Supplementary Fig. 6a the same method as shown above for the intermediate Γ regime.

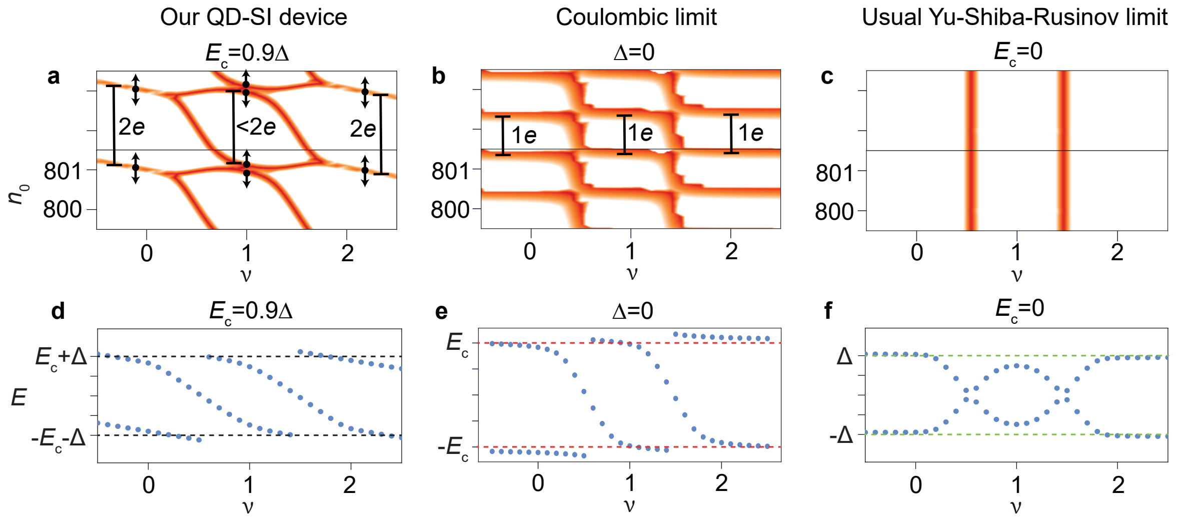

Supplementary Fig. 9. **Spectral comparisons with Coulombic and usual Yu-Shiba-Rusinov (YSR) limits.** (**a**-**c**) Calculated diagram of GS transitions (collage of two identical plots) versus the gate-induced charge in the QD, ν, and the gate-induced charge in the SI, n_0, for (**a**) our QD-SI device parameters, (**b**) Coulombic limit ($\Delta = 0$), and (**c**) YSR limit ($E_\mathrm{c} = 0$). Bars indicate the vertical size of charge domains in electron charge units. Whereas in (**a**) Δ modulates this size depending on ν and favors $2e$ charging away from $\nu \approx 1$, in (**b**) the size is strictly $1e$ independently of ν and a honeycomb pattern is obtained. Arrows in (**a**) are anchored to GS transition lines which split when the ratio E_c/Δ is increased. In (**c**), absence of charging results in no GS dependence on n_0. (**d**-**f**) Calculated first-excitation spectrum versus ν for $n_0 = 800$ for each of the cases shown in (**a**-**c**). Dashed lines of different color highlight the position of the different energy gaps in each case: (**d**) $E_\mathrm{c} + \Delta$, (**e**) E_c, and (**f**) Δ. Aside from this difference, since E_c is sizeable in our device, the spectrum in (**d**) resembles qualitatively the Coulombic limit in (**e**). However, due to the presence of an equally sizeable Δ in our device, the differences are very marked near $n_0 = 801$, as shown for the GS in (**a**, **b**). The energy-asymmetric and discontinuous spectra in (**d**, **e**) are to be contrasted with the usual YSR limit in (**f**). Parameters for (**a**, **d**) are noted in Table 1. For (**b**, **e**): $U/E_\mathrm{c} = 4$, $\Gamma/U = 0.05$, $V = 0$. For (**c**, **f**): $U/\Delta = 4$, $\Gamma/U = 0.05$, $V = 0$. $N = 800$ in all cases.

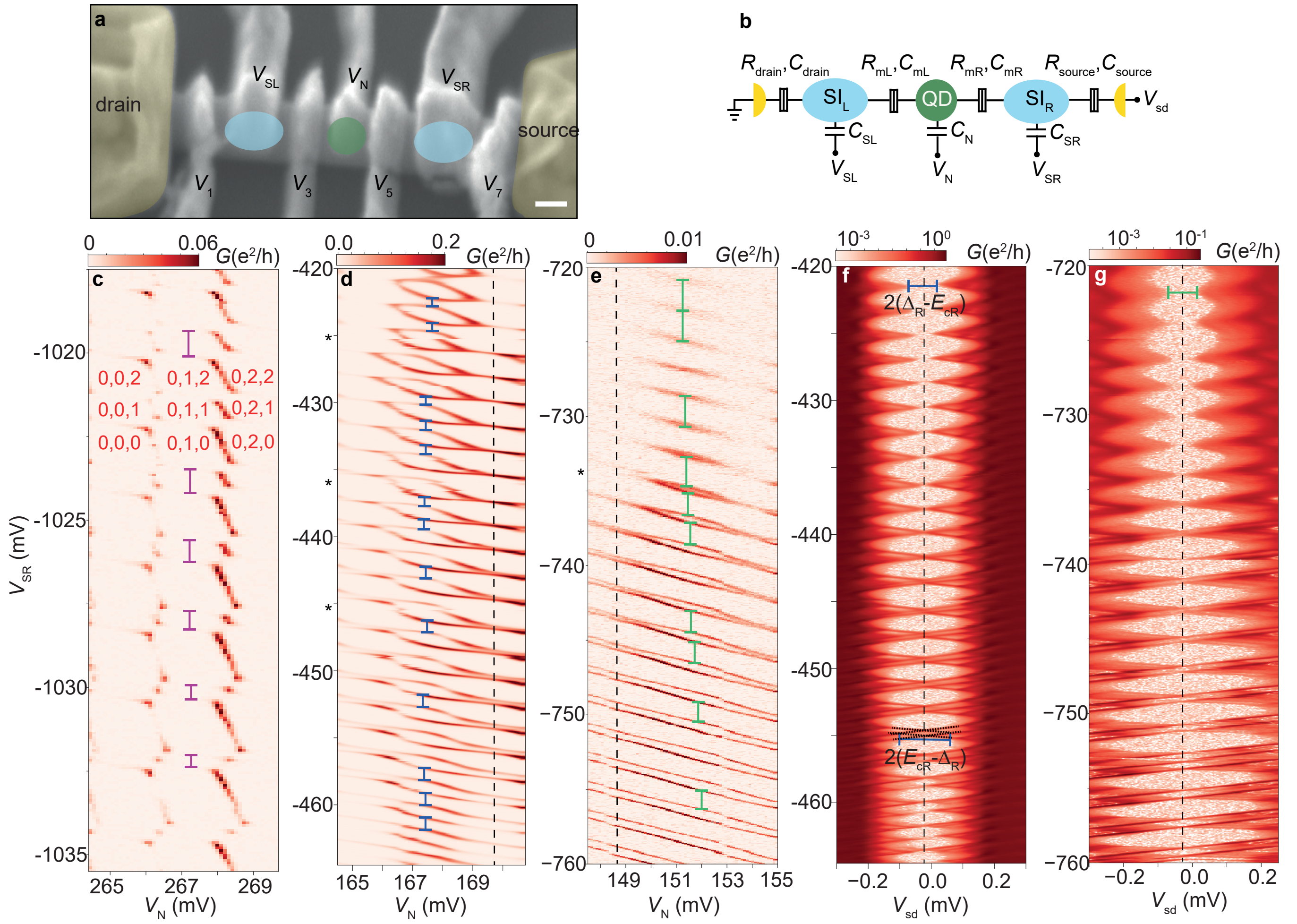

Supplementary Fig. 10. **Tuning the Coulomb repulsion in the two-island device.** **a** Scanning electron micrograph of the SI-QD-SI device, comprising an InAs nanowire with Al SIs (below top gates SL and SR). Top gates 3 and 5 confine a QD below top gate N. Top gates 1 and 7 are shorted to top gates SL and SR, respectively. Scale bar is 100 nm. **b** Electrostatics of the device. **c, d, e** Zero-bias G versus gate voltage of the right SI, V_{SR}, and gate voltage of the QD, V_{N}, from which the data plotted in Fig. 4 (indicated here by vertical bars) was extracted. Other gates are set to (**c**) $V_{\mathrm{SL}} = -761$ mV, $V_3 = -505$ mV, $V_5 = -330$ mV, $V_{\mathrm{bg}} = -0.05$ V, (**d**) $V_{\mathrm{SL}} = -536$ mV, $V_3 = -443$ mV, $V_5 = -170$ mV, $V_{\mathrm{bg}} = -2.69$ V, and (**e**) $V_{\mathrm{SL}} = -260$ mV, $V_3 = -830$ mV, $V_5 = 150$ mV, $V_{\mathrm{bg}} = -0.48$ V, with V_5 controlling Γ_{R} by accumulating electrons in the nanowire. V_{SL} corresponds to n_{SL} =even, where the left SI does not play a role in defining the GS. Approximate charges in the parts of the device (n_{SL},n_{N},n_{SR}) are indicated. Tuned by V_{SR}, $E_{\mathrm{cR}} - \Delta_{\mathrm{R}}$ increases progressively from bottom to top in (**c**) and from top to bottom in (**d**, **e**), expanding $n_{\mathrm{SR}} = 1$ sectors. Charge sectors are identified by the approximately integer occupations in the three Coulomb-blockaded parts of the device (n_{SL},n_{N},n_{SR}) indicated in red. The singlet assignment in (**e**) is verified by the B dependence of the stability diagram (see Supplementary Fig. 11). **f**, **g** G versus V_{SR} and source-drain bias voltage, V_{sd}, with V_{SR} swept along the dashed line in (**d**), where $n_{\mathrm{QD}} = 2$, and in (**e**), where $n_{\mathrm{QD}} = 0$. The true zero bias (indicated by a dashed line) is offset by $V_{\mathrm{sd}} = -18\ \mu$V. When $E_{\mathrm{cR}} - \Delta_{\mathrm{R}} < 0$, the vertices of the Coulomb diamonds do not touch zero bias, while for $E_{\mathrm{cR}} - \Delta_{\mathrm{R}} > 0$ small diamonds emerge. Bars indicate how $E_{\mathrm{cR}} - \Delta_{\mathrm{R}}$ relates to these features. Asterisks denote gate glitches.

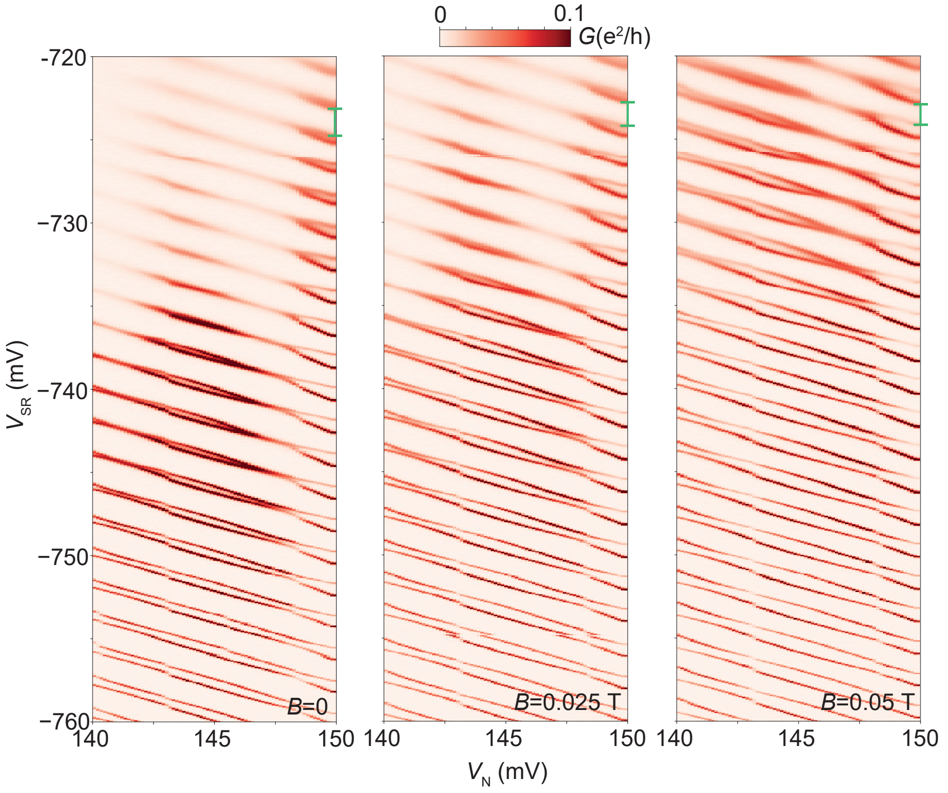

Supplementary Fig. 11. **Corroboration of the singlet assignment in Supplementary Fig. 10e.** Zero-bias G versus gate voltage of the right SI, $V_{\rm SR}$, and gate voltage of the QD, $V_{\rm N}$, at different magnetic field B values. Other gates are set as in Supplementary Fig. 10e. Green bars highlight the expected reduction with B of singlet domains in their $V_{\rm SR}$ extension, which corroborates their spin assignment.

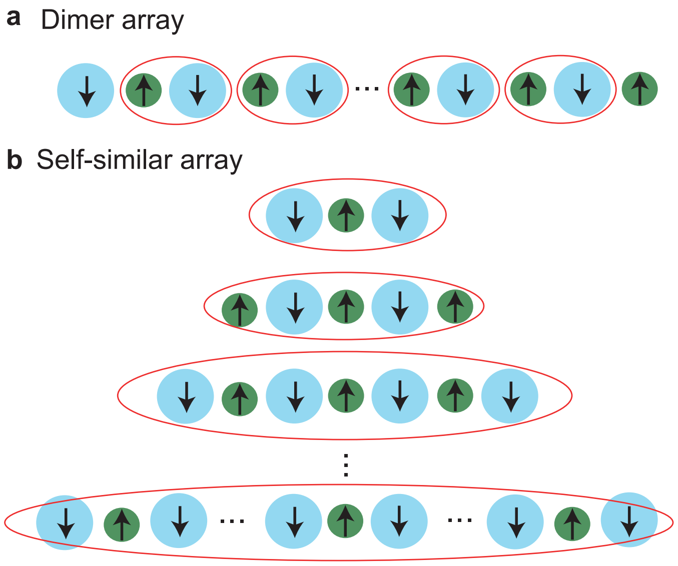

Supplementary Fig. 12. **Possible extensions of the QD-SI and SI-QD-SI devices. a** Array of QD-SI dimers, with each QD and SI having an electron and quasiparticle, respectively. The array has an even number of elements. The SI and QD at the ends of the array are not part of a dimer. Instead, they form a long-range singlet. **b** Recursive screening of a SI-QD-SI array. The arrays have an odd number of elements, with two end elements added in each iteration. The arrays simulate the intermediate coupling fixed point of the two channel Kondo model when they incorporate a sufficiently large number of elements. The ground state of these arrays is an overscreened doublet with long-range triplet correlations between the end spins. The correlations are maximized when the Yu-Shiba-Rusinov antiferromagnetic binding interaction is (**a**) strongly asymmetric between a given spin and its left and right nearest neighbors, or (**b**) symmetric between any spin and its closest neighbors.

Supplementary Note 3. Model of the quantum dot - superconducting island system

The QD is described as a single non-degenerate impurity level, as in the single-impurity Anderson model (SIAM). The SI is described as a set of equidistant energy levels that represent time-reversal-conjugate pairs in the momentum/orbital space, coupled all-to-all by the pairing interaction. This step beyond the BCS mean-field approximation is required to accurately describe the strong even-odd occupancy effects of the SI, which arise from its large charging energy E_c. The QD is coupled to all levels of the SI via a hybridisation term. The Hamiltonian used to model the system is

$$\begin{aligned} H_{\mathrm{QD}} &= \varepsilon_{\mathrm{QD}}\hat{n}_{\mathrm{QD}} + U\hat{n}_{\mathrm{QD},\uparrow}\hat{n}_{\mathrm{QD},\downarrow} + E_{Z,\mathrm{QD}}\hat{S}_{z,\mathrm{QD}} \\ &= \frac{U}{2}(\hat{n}_{\mathrm{QD}} - \nu)^2 + E_{Z,\mathrm{QD}}\hat{S}_{z,\mathrm{QD}} + \mathrm{const.}, \end{aligned} \tag{1}$$

$$\begin{aligned} H_{\mathrm{SC}} = \sum_{i,\sigma}^{N} \varepsilon_{\sigma,i} c^\dagger_{i,\sigma} c_{i,\sigma} - \alpha d \sum_{i,j} c^\dagger_{i,\uparrow} c^\dagger_{i,\downarrow} c_{j,\downarrow} c_{j,\uparrow} \\ + E_c(\hat{n}_{\mathrm{SC}} - n_0)^2, \end{aligned} \tag{2}$$

$$\begin{aligned} H_{\mathrm{hyb}} = \frac{v}{\sqrt{N}} \sum_{i,\sigma} \left(c^\dagger_{i,\sigma} d_\sigma + \mathrm{h.c.} \right) \\ + V(\hat{n}_{\mathrm{QD}} - \nu)(\hat{n}_{\mathrm{SC}} - n_0), \end{aligned} \tag{3}$$

$$H = H_{\mathrm{QD}} + H_{\mathrm{SC}} + H_{\mathrm{hyb}}. \tag{4}$$

Here d_σ and $c_{i,\sigma}$ are annihilation operators of the QD and the bath, $\sigma = \uparrow, \downarrow$, $\hat{n}_{\mathrm{QD},\sigma} = d^\dagger_\sigma d_\sigma$ are impurity occupancy operators, $\hat{n}_{\mathrm{QD}} = \sum_\sigma \hat{n}_{\mathrm{QD},\sigma}$, and $\hat{S}_{z,\mathrm{QD}} = (\hat{n}_{\mathrm{QD},\uparrow} - \hat{n}_{\mathrm{QD},\uparrow})/2$ is the impurity spin operator in the direction of the external magnetic field. $\varepsilon_{\mathrm{QD}}$ is the energy of the impurity level, U the electron repulsion, $E_{Z,\mathrm{QD}} = g_{0N}\mu_B B$ is the impurity Zeeman energy, where g_{0N} is the corresponding bare g-ratio, μ_B is the Bohr magneton, and B the magnetic field, while $\nu = \frac{1}{2} - \frac{\varepsilon_{QD}}{U}$ is the energy level in units of electron number.

The superconductor energy levels ε_i are spaced by $d = 2D/N$, where the index $i = 1, \ldots, N$, D is the half-bandwidth and N is the number of levels. α is the strength of the pairing interaction, E_C is the charging energy and n_0 is the gate voltage applied to the SI, expressed in the units of electron number. The number of electrons in the island is $\hat{n}_{\mathrm{SC}} = \sum_{i,\sigma} c^\dagger_{i,\sigma} c_{i,\sigma}$. The SI Zeeman energy is incorporated in the energy levels as $\varepsilon_{\sigma,i} = \varepsilon_i + n_\sigma E_{Z,\mathrm{SI}}/2$ where $n_\uparrow = 1$ and $n_\downarrow = -1$, $E_{Z,\mathrm{SI}} = g_{0S}\mu_B B$.

The hybridisation strength is $\Gamma = \pi\rho v^2$, where $\rho = 1/2D$ is the normal state bath density of states. The V term describes the capacitive coupling between the QD and the SI, which was found to be important to correctly reproduce the charging diagrams of the device studied in this work. We take the half-bandwidth $D = 1$ as the unit of energy.

The hybridisation strengths of SI and QD with the neighboring lead, Γ_{source} and Γ_{drain}, respectively, do not enter the Hamiltonian directly. They are assumed weak and their small effect on the system can be accounted through a small renormalization of other parameters.

For exploring the vast parameter space of the model, we found it useful to perform quick simulations with a significantly smaller system size of $N = 20$ and a larger value of $\alpha = 0.4$, so that $\Delta \approx 0.16D$. To compensate for the very large finite-size effects in this case, we corrected the resulting eigenvalues by subtracting the product of $d/2$ and the absolute value of the excess charge in the superconductor (for excess charge less than 2 in absolute value). This correction procedure works surprisingly well. Further improvement is possible by averaging even-N and odd-N results since the $N \to \infty$ limit is approached from different sides [9], leading to a significant cancellation of finite-size effects.

Highly converged simulation results that we used for direct comparisons with the experimental measurements in the main text of this work were performed for a very large number of bath levels, $N = 800$. This value is large enough to minimize the finite-size effects even without finite-size corrections. With finite-size corrections, equivalent results can be obtained for significantly smaller system size, $N = 200$. We set $\alpha = 0.23$, a magnitude appropriate for Al grains [10], which determines the superconducting gap in the absence of impurity, $\Delta = 0.026D$.

The calculations were performed using the DMRG as described in Supplementary Ref. [9] with three modifications of the matrix-product-operator (MPO) expression of the Hamiltonian: 1) incorporation of the QD-SI capacitive coupling $V(\hat{n}_{\mathrm{QD}} - \nu)(\hat{n}_{\mathrm{SC}} - n_0)$, 2) addition of the impurity Zeeman term $E_{Z,\mathrm{QD}}$, 3) addition of the bath Zeeman term $E_{Z,\mathrm{SI}}$. The full expression of the MPO is as follows (notation follows that of Supplementary Ref. [9]). Left-most site (impurity-site):

$$W_0 = \begin{pmatrix} I & h_{\mathrm{imp}} & -d_\uparrow F & -d_\downarrow F & +d^\dagger_\uparrow F & +d^\dagger_\downarrow F & 0 & 0 & V\hat{n}_{\mathrm{imp}} \end{pmatrix} \tag{5}$$

Here $h_{\mathrm{imp}} = (\epsilon_{\mathrm{imp}} - Vn_0)\hat{n}_{\mathrm{imp}} + U\hat{n}_{\mathrm{imp},\uparrow}\hat{n}_{\mathrm{imp},\downarrow} + E_{Z,\mathrm{QD}}\hat{S}_{z,\mathrm{QD}}$ and $F = (-1)^n$ is the local fermionic-parity operator, which gives a phase of -1 if there is an odd number of electrons on the site.

Generic site (with $g = \alpha d$):

$$W_i = \begin{pmatrix} 1 & h_i & 0 & 0 & 0 & 0 & g c_{i\downarrow} c_{i\uparrow} & g c^\dagger_{i\uparrow} c^\dagger_{i\downarrow} & 2E_c\hat{n}_i \\ 0 & I & 0 & 0 & 0 & 0 & 0 & 0 & 0 \\ 0 & v c^\dagger_{i\uparrow} & F_i & 0 & 0 & 0 & 0 & 0 & 0 \\ 0 & v c^\dagger_{i\downarrow} & 0 & F_i & 0 & 0 & 0 & 0 & 0 \\ 0 & v c_{i\uparrow} & 0 & 0 & F_i & 0 & 0 & 0 & 0 \\ 0 & v c_{i\downarrow} & 0 & 0 & 0 & F_i & 0 & 0 & 0 \\ 0 & c^\dagger_{i\uparrow} c^\dagger_{i\downarrow} & 0 & 0 & 0 & 0 & I & 0 & 0 \\ 0 & c_{i\downarrow} c_{i\uparrow} & 0 & 0 & 0 & 0 & 0 & I & 0 \\ 0 & \hat{n}_i & 0 & 0 & 0 & 0 & 0 & 0 & I \end{pmatrix}, \tag{6}$$

with $h_i = [\epsilon_i - V\nu + E_c(1 - 2n_0)]\hat{n}_i + E_{Z,\mathrm{SI}}\hat{S}_{z,i} + (g + 2E_c)\hat{n}_{i\uparrow}\hat{n}_{i\downarrow}$, where $\hat{S}_{z,i} = (\hat{n}_{i\uparrow} - \hat{n}_{i\downarrow})/2$.

Right-most site:

$$W_N = \begin{pmatrix} h_N \\ I \\ v c^{\dagger}_{N\uparrow} \\ v c^{\dagger}_{N\downarrow} \\ v c_{N\uparrow} \\ v c_{N\downarrow} \\ c^{\dagger}_{N\uparrow} c^{\dagger}_{N\downarrow} \\ c_{N\downarrow} c_{N\uparrow} \\ \hat{n}_N \end{pmatrix}. \tag{7}$$

[1] J. M. Hergenrother, M. T. Tuominen, and M. Tinkham, "Charge transport by Andreev reflection through a mesoscopic superconducting island," Phys. Rev. Lett. **72**, 1742–1745 (1994).

[2] W. G. van der Wiel, S. De Franceschi, J. M. Elzerman, T. Fujisawa, S. Tarucha, and L. P. Kouwenhoven, "Electron transport through double quantum dots," Rev. Mod. Phys. **75**, 1–22 (2002).

[3] J. C. Estrada Saldaña, A. Vekris, G. Steffensen, R. Žitko, P. Krogstrup, J. Paaske, K. Grove-Rasmussen, and J. Nygård, "Supercurrent in a Double Quantum Dot," Phys. Rev. Lett. **121**, 257701 (2018).

[4] J. C. Estrada Saldaña, A. Vekris, R. Žitko, G. Steffensen, P. Krogstrup, J. Paaske, K. Grove-Rasmussen, and J. Nygård, "Two-impurity Yu-Shiba-Rusinov states in coupled quantum dots," Phys. Rev. B **102**, 195143 (2020).

[5] Juan Carlos Estrada Saldaña, Alexandros Vekris, Victoria Sosnovtseva, Thomas Kanne, Peter Krogstrup, Kasper Grove-Rasmussen, and Jesper Nygård, "Temperature induced shifts of Yu–Shiba–Rusinov resonances in nanowire-based hybrid quantum dots," Commun. Phys. **3**, 1–11 (2020).

[6] Andrey E. Antipov, Arno Bargerbos, Georg W. Winkler, Bela Bauer, Enrico Rossi, and Roman M. Lutchyn, "Effects of Gate-Induced Electric Fields on Semiconductor Majorana Nanowires," Phys. Rev. X **8**, 031041 (2018).

[7] A. C. Johnson, J. R. Petta, C. M. Marcus, M. P. Hanson, and A. C. Gossard, "Singlet-triplet spin blockade and charge sensing in a few-electron double quantum dot," Phys. Rev. B **72**, 165308 (2005).

[8] B. van Heck, R. M. Lutchyn, and L. I. Glazman, "Conductance of a proximitized nanowire in the Coulomb blockade regime," Phys. Rev. B **93**, 235431 (2016).

[9] Luka Pavešić, Daniel Bauernfeind, and Rok Žitko, "Subgap states in superconducting islands," Phys. Rev. B **104**, L241409 (2021).

[10] Fabian Braun and Jan von Delft, "Fixed-n superconductivity: The crossover from the bulk to the few-electron limit," Physical Review Letters **81**, 4712–4715 (1998).
